# Development and mineralization of embryonic avian scleral ossicles

**Published:** 2012-02-05

**Authors:** Guodong Zhang, Daniel L. Boyle, Yuntao Zhang, Austin R. Rogers, Gary W. Conrad

**Affiliations:** Division of Biology, Kansas State University, Manhattan, KS

## Abstract

**Purpose:**

To investigate the development and mineralization of avian scleral ossicles using fluorescence microscopy in combination with field emission scanning electron microscopy (FESEM) and energy dispersive spectroscopy (EDS).

**Methods:**

The anterior halves of whole eyeballs from chickens on embryonic (E) days E10 to E21 and Japanese quail on embryonic days E8 to E17 were fixed in 100% methanol for 1 min, stained with Giemsa solution for 5 min, destained with distilled water for 30 min, and then viewed by epifluorescence. Propidium iodide (PI) was used to detect the nuclei of osteocytes in scleral ossicles. FESEM and EDS were then used to show areas of mineralization and to identify differences in the elemental composition of different regions of the ossicles.

**Results:**

Using Giemsa as a fluorescence stain, it was possible to observe the detailed morphology and development of both chicken and quail scleral ossicles. In chickens, bone microporosities first became visible at E15. Each microporosity contained a single nucleus, likely that of an osteocyte. The amount of carbon in ossicles steadily decreased during embryogenesis and post-hatching, while the concentration of oxygen showed a distinct increase over this time period. Calcium and phosphate levels in the ossicles increased gradually during embryonic and post-hatching stages.

**Conclusions:**

A novel approach to study the development and mineralization of avian scleral ossicles during embryogenesis is presented. This methodology was validated by studying two different species, both important models for avian developmental research.

## Introduction

Scleral ossicles are rings of overlapping trapezoid-shaped membrane bones, which are embedded in the sclera surrounding the cornea beneath the conjunctival zone in the eyes of non-mammalian vertebrates [1-3]. The biologic function of the scleral ossicle ring as a whole, as well as that of the individual ossicles, is poorly understood. It is often proposed that the scleral ring provides protection against pressure changes and helps maintain the shape of the cornea [4]. In addition, scleral ossicles may act as a point of attachment for the ciliary muscles, specifically the anterior ciliary muscle, suggesting a role in corneal accommodation [5-7]. The development, number, arrangement and position of the bony plates forming the ring show slight differences between distinct groups of vertebrates. For bird species, the number of bony plates varies from 11 to 16, with most having 14 in each scleral ossicle ring. These bony plates usually have a quadrangular shape and similar arrangement patterns are seen in birds in the same taxonomic order [8-10].

To date, the morphology and development of scleral ossicles have only been studied by bright-field microscopy, based on Alizarin Red S staining of ossified material in whole mount preparations of small vertebrates [4,8-11]. Alizarin Red S is an anthraquinone derivative that reacts with tissue calcium to form an Alizarin Red S-calcium complex in a chelation process producing a birefringent end product [12,13]. The reaction is not strictly specific to calcium and interference from magnesium, albumin, manganese, and iron may impede results [14]. However fluorescence microscopy, which uses a calcium-sensitive dye, is a rapid, sensitive method that is well suited for bone staining [15].

Giemsa is a classic stain for bone marrow, gastric tissue, and peripheral blood smears [16]. Giemsa solution is a mixture of methylene blue, eosin, and azure B. Interestingly, a previous study suggested that Giemsa could be used as a fluorescent stain to identify mineralized bone using epifluorescence microscopy [16]. The fluorescent properties of Giemsa are due to eosin Y, one of its components, which shows an emission peak centered at 550 nm after excitation with 490 nm light [17], suggesting that it may be useful for studying the morphological changes in avian scleral ossicles.

To help maintain the shape of the cornea, it is important for scleral ossicles to be mineralized and hard, with high plate strength. Bone strength is determined not only by the volume of bone tissue and its microarchitectural organization, but also by the degree of mineralization in the bone matrix [18,19]. Mineralization of bone involves a well orchestrated process in which crystals of calcium phosphate are produced by bone-forming cells (osteocytes) and laid down in precise amounts within the fibrous matrix or scaffolding of the bone [20]. However, the process of mineralization of scleral ossicles during development has not been elucidated. In this study, fluorescence microscopy, field emission scanning electron microscopy (FESEM) and energy dispersive spectroscopy (EDS) were employed to characterize the development and mineralization of avian scleral ossicles.

## Methods

### Embryo development and isolation of the anterior half of the eye

All animals were used in accordance with the ARVO Statement for the Use of Animals for Ophthalmic and Vision Research. Fertilized eggs from White Leghorn chickens were purchased from Nelson Poultry Farms Inc. (Manhattan, KS) and fertilized Japanese quail eggs were purchased from B&D Game Farm (Harrah, OK). Before shipping to our laboratory, both sets of eggs were collected and stored at 15 °C for 1 to 3 days after laying. Both types of eggs were stored at 15 °C for no more than a week after arriving at our laboratory. These fertilized eggs were incubated at 38 °C and 45% humidity from embryonic day (E) 0, the day on which the eggs were put into the incubator. The anterior half of the eye was dissected from chicken eggs at embryonic ages E10 to E21 and from quail eggs from E8 to E17 and rinsed in sterile PBS (phosphate buffered saline, 0.1 M sodium phosphate, 0.15 M sodium chloride, pH 7.2). Before staining, adherent tissues on the surface of scleral ossicles were gently removed using a blunt knife. The lens and iris were then removed.

### Staining

Immediately after dissection, the anterior half of each eyeball, consisting of only the cornea and surrounding limbus, the conjunctiva, and the sclera, was fixed for 1 min in 100% methanol at room temperature, with mild agitation, and then immediately stained in Giemsa solution (azure eosin methylene-blue solution; PN 295591000; Fisher Scientific, Hanover Park, IL) for 5 min. The tissues were subsequently destained with distilled water for 30 min. Throughout this process, the samples were kept in constant gentle movement with a rotary shaker set at 1 revolution/s. while wrapped in aluminum foil to prevent photo bleaching and loss of fluorescence. To compare with Giemsa staining, the anterior halves of separate chicken eyeballs were stained with Alizarin Red S. In the current study, the Alizarin Red S staining protocol was modified from the procedure described previously [4,8]. Briefly, the anterior half of the eyeball was dissected from chicken eggs at embryos ages E10 to E21, fixed in 10% formaldehyde solution overnight at room temperature, and then stained with 1% Alizarin Red S in distilled water for 10 min. The stained tissues were destained with distilled water for 3 h, and imaged using the bright-field of a stereomicroscope (Leica MZ16F, Wetzlar, Germany).

### Stereomicroscope imaging

After staining, the anterior halves of chicken and quail eyeballs were transferred to glass slides with the cornea facing down, covered with a coverslip, and imaged using a fluorescence stereomicroscope (Leica MZ16F) equipped with filters (Leica GFP2, 480 nm excitation filter/510 nm barrier filter).

### Confocal laser scanning microscopy

The chicken scleral ossicles were imaged using a Carl Zeiss LSM700 microscope (Carl Zeiss Microscope Systems, Oberkochen , Germany) with the following parameters: excitation with a 488 nm laser line from an argon ion LASER, and a BP 505–530 nm emission filter. Plan-Neofuar 40×/1.3 DIC objective with the airy unit set to 1 and an optical slice interval of 1.7 µm.

### Detecting osteocytes

The anterior halves of chicken anterior eyeballs (E16 and E20) stained with Giemsa, as described above, were then stained with propidium iodide (PI; Invitrogen, Carlsbad, CA). Briefly, a 5 µM PI staining solution was made by diluting the 1 mg/ml (1.5 mM) stock solution in 2× SSC (0.3 M NaCl, 0.03 M sodium citrate, pH 7.0). After the Giemsa destaining step, the anterior halves of the chicken eyes were incubated in the PI solution (200 µl) for 30 min at room temperature, rinsed twice with SSC for 1 min, and then viewed by confocal microscopy (Carl Zeiss LSM700 system) [21]. The parameters used were: a 488 nm laser line from an argon ion LASER, a 543 nm laser line from a HeNe LASER, HFT 488/543 nm, NFT 545 nm, BP 505–530 nm emission filter (Giemsa), and LP 585 nm emission filter (PI). Z-series through ossicles were collected with a Plan-Neofuar 40×/1.3 DIC objective with the airy unit set to 1 and an optical slice interval of 1.7 µm.

### FESEM and energy dispersive spectroscopy (EDS) analysis

Individual trapezoid-shaped ossicles were dissected from the eyes using a stereomicroscope and freed from other adherent tissues. For cross-sectional imaging and analysis, individual ossicles were bisected parallel to the corneal-scleral interface plane. Ossicles were oriented and mounted on aluminum SEM stubs with carbon adhesive tape to image and analyze the ossicle surface or cross-sections. Imaging and elemental analysis were performed using a field emission scanning electron microscope, Nova NanoSEM 430 (FEI, Hillsboro, OR) equipped with an X-Max Large Area Analytical energy dispersive spectroscopy (EDS) silicon drift detector (SDD; 80 mm^2^; Oxford Instruments, Bucks, United Kingdom). For EDS, the accelerating voltage was 15 kV, the spot size was 4.5 (0.70 nA), and data were collected over 120 s. Silicon was used for quantum optimization of the EDS. The weight percent (Wt %) of elements were directly calculated and given by the Nova NanoSEM 430 system when analyzing the ossicles.

### Statistical analysis

Data are presented as means±standard deviation (SD) from three separate experiments. Statistical analyses were performed using Student’s *t*-tests to compare readings from the surface and the interior of the ossicles, assuming equal variances. A p-value less than 0.05 was considered to be significant.

## Results

**Figure 1:**
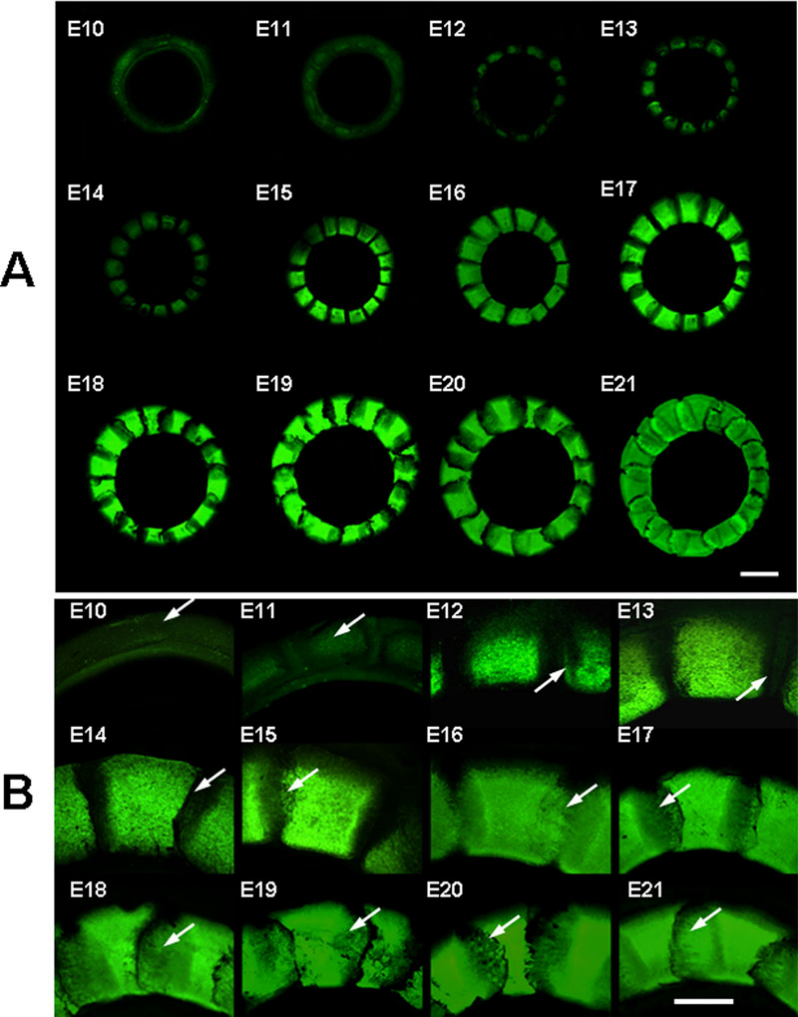
The morphology of developing chicken scleral ossicles imaged using fluorescence stereomicroscopy. **A**: The development of chicken scleral ossicles at stages E10 to E21. **B**: The details of ossification, including the overlapping of adjacent ossicles along the dorsal quadrant of the ring. **A**: Scale bar: 1 mm; **B**: Scale bar: 0.5 mm.

**Figure 2:**
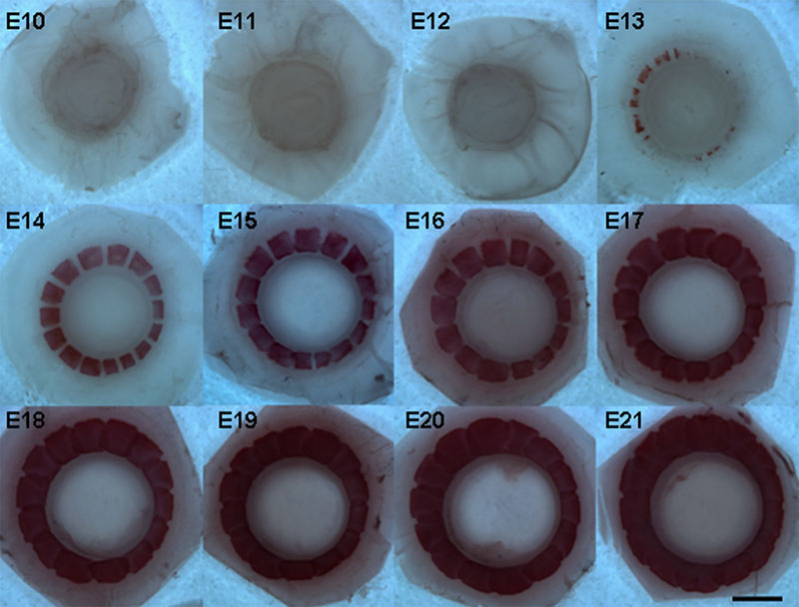
The patterns of developing chicken scleral ossicles at stages E10 to E21 stained with Alizarin Red S. Scale bar: 1 mm.

Using the Giemsa staining protocol established here, the developmental patterns of both chicken and quail embryonic scleral ossicles were visualized by fluorescence microscopy. Figure 1A,B show the development of chicken embryonic scleral ossicles from E10 to E21. As evident in Figure 1B, prominent preossicular condensations were detected as early as E10 (arrow) and the first signs of ossification were present by E11 (arrow, Figure 1A,B). By E12, all ossicle plates were being formed (Figure 1A). Overt osteogenesis, as detected with this Giemsa technique, appeared at E13 (arrow, Figure 1B), with edges of enlarging plates beginning to overlap at E15 (arrow, Figure 1B) and full overlapping to form the complete ossicle ring achieved by E17. The ossicle ring persisted until hatching on E21 (Figure 1A,B). The images in Figure 1B show the boney plate interactions and progressive overlapping only in the dorsal quadrant of the ossicle ring. Correspondingly detailed images from the other quadrants are shown in Appendix 1 (**A**: temporal; **B**: nasal; and **C**: ventral). Finally, to allow comparison of these Giemsa images with those from an independent bone stain technique, chicken scleral ossicles also were stained with Alizarin Red S (Figure 2). One of several advantages of the Giemsa technique is that it allows earlier detection of ossicle plates (Giemsa: Figure 1: E12; Alizarin Red S: Figure 2: E14).

**Figure 3:**
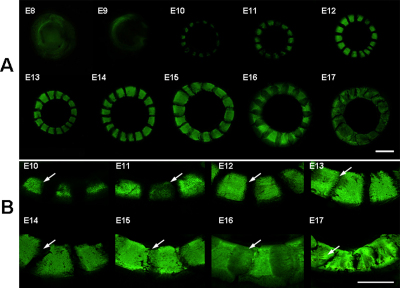
The morphology of developing Japanese quail scleral ossicles imaged using fluorescence stereomicroscopy. **A**: The development of quail scleral ossicles at stages E8 to E17. **B**: The details of ossification, including the overlapping of adjacent ossicles. **A**: Scale bar: 1 mm; **B**: Scale bar: 0.5 mm.

Figure 3A,B shows the development of Japanese quail scleral ossicles from E8 to E17 as revealed by Giemsa staining. Prominent preossicular condensations formed as early as day E8 (Figure 3A) and the first sign of ossification appeared on E9 (Figure 3A). Osteogenesis was detected during E10 (Figure 3A) and the full set of ossicle plates could be seen by E11 (Figure 3A). The ossicle plates started to overlap during days E13-E14 (arrow, Figure 3B), and the complete ossicle ring had formed by E15 (arrow), which persisted until hatching on E17 (Figure 3A,B).

**Figure 4:**
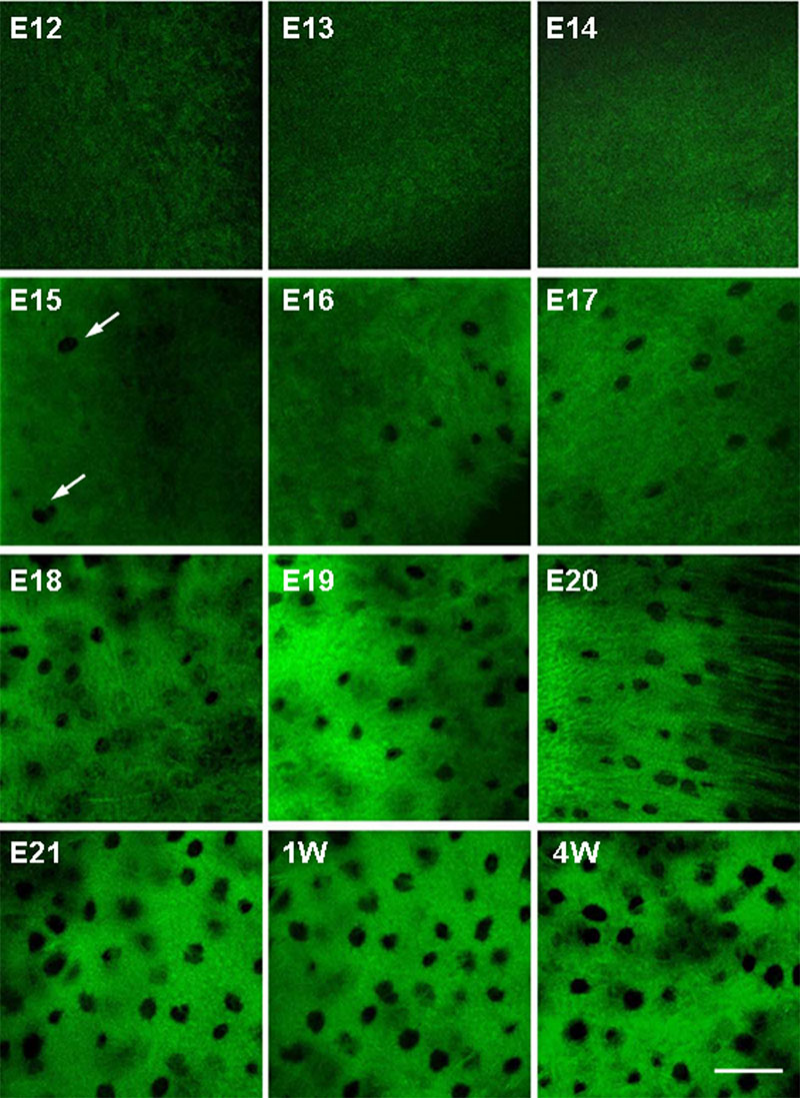
Representative confocal slices of Z-series, showing bone microporosities in chicken scleral ossicles. The bone microporosities became visible by E15 (arrow) and their numbers increase for a given field of view with increasing development of the ossicle plate (E15 to E21). Scale bar: 50 µm.

**Figure 5:**
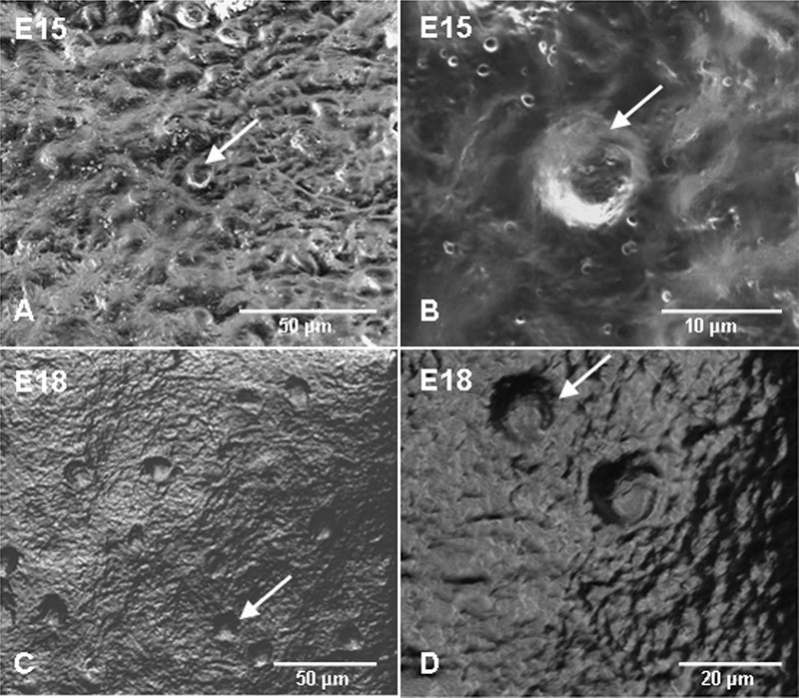
FESEM images showing the bone microporosities in the surface of chicken scleral ossicles. **A** and **B**: Features of the bone microporosities of E15 chicken scleral ossicles (arrow). **C** and **D**: Patterns of the bone microporosities of E18 chicken scleral ossicles (arrow).

**Figure 6:**
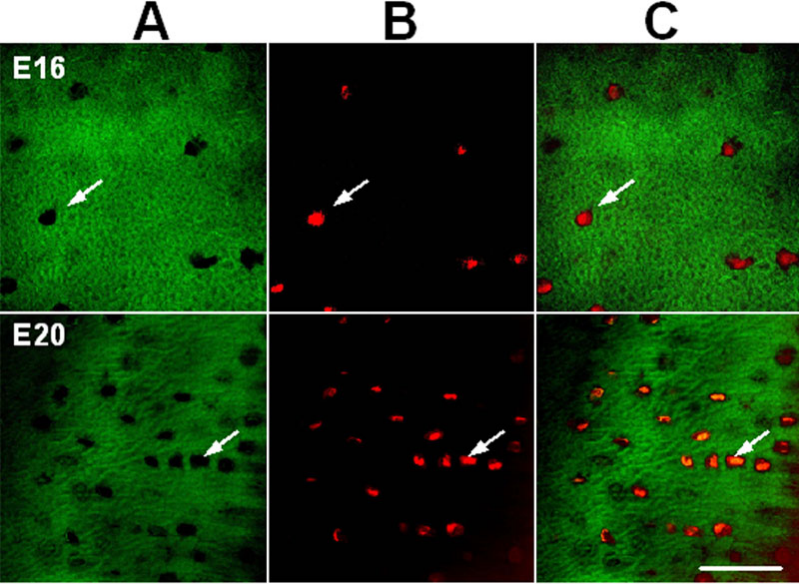
Representative confocal slices of Z-series showing Giemsa and PI staining in chicken ossicles. The whole anterior halves of eyes from chicken embryos were stained with both Giemsa and propidium iodide. **A**: Patterns of the microporosities of chicken scleral ossicles (E16 and E20). **B**: Features of nuclei in chicken scleral ossicles (E16 and E20). **C:** Profiles of the ossicle microporosities containing nuclei, indicating that an individual nucleus resides in each bone microporosity (arrows). Scale bar, 50 µm.

Representative confocal slices of single chicken scleral ossicles during different stages of development are displayed in Figure 4. Arrays of microporosities were observed by E15 (arrows) and the density per given field of view increased during the development of the ossicle plate (Figure 4). The details of the bone microporosities of chicken scleral ossicles (E15 and E18) were displayed with FESEM (Figure 5), supporting the confocal data in Figure 4 and Figure 6. Confocal slices of single chicken ossicles at different developmental stages showed that PI-stained nuclei could be observed within microporosities on days E16 and E20 (Figure 6), suggesting that an individual osteocyte resides in each single bone microporosity.

**Figure 7:**
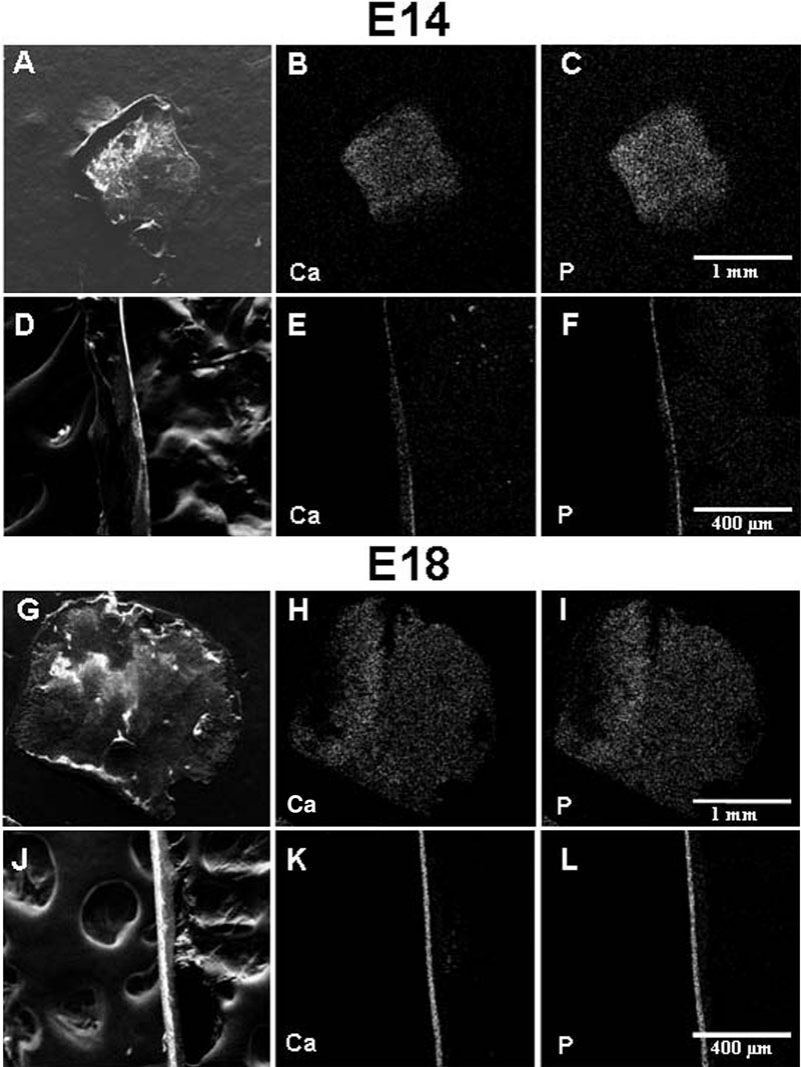
Representative FESEM EDS mapping analysis of chicken ossicles. Surface (**A**, **G**) and cross sectional (**D**, **J**) secondary electron images and corresponding calcium (**B**, **E**, **H**, and **K**) and phosphorus (**C**, **F**, **I**, and **L**) EDS maps are presented from two different developmental time periods: E14 (**A**, **B**, **C**, **D**, **E**, and **F**) and E18 (**G**, **H**, **I**, **J**, **K**, and **L**). In maps, white pixels indicate both the intensity (quantity) and location of the given element.

Results of FESEM and EDS mapping analysis indicated that concentrations of calcium (Ca) and phosphorus (P) on the surface of embryonic chicken ossicles were distinctly lower than those in the interior region detected in cross sections (Figure 7). Data collected from a minimum of four different ossicles at each stage of development (E14, E16, and E18) are summarized in Table 1, indicating that the concentrations of Ca and P at the surface of chicken ossicles were distinctly lower than in the interior region detected in cross sections.

**Table 1:** Concentration levels for the main elements detected on the surface and in the interior of chicken embryonic scleral ossicles.

**Embryonic age**	**Region of the ossicle**	**C**	**O**	**Ca**	**P**
E14	Surface	60.39±0.58	36.83±0.86	1.33±0.17	1.21±0.13
	Interior	55.45±3.85*	34.93±4.68	5.50±1.64**	3.58±0.36**
E16	Surface	58.87±1.53	36.97±0.52	1.91±0.43	1.90±0.42
	Interior	44.69±1.31**	42.29±1.00**	7.04±0.82**	4.45±0.40**
E18	Surface	53.31±0.11	40.30±0.37	3.12±0.11	3.23±0.10
	Interior	43.46±3.85*	43.44±1.99*	7.72±1.43**	5.09±0.81*

* Indicates a significant difference (p<0.05) between concentrations on the surface and in the interior. ** Indicates a highly significant difference (p<0.01) between concentrations on the surface and in the interior. Concentration levels of the main elements are the percentage of total elements detected by weight. Each value is expressed as the mean±SD from three repeated samples (n=3).

**Figure 8:**
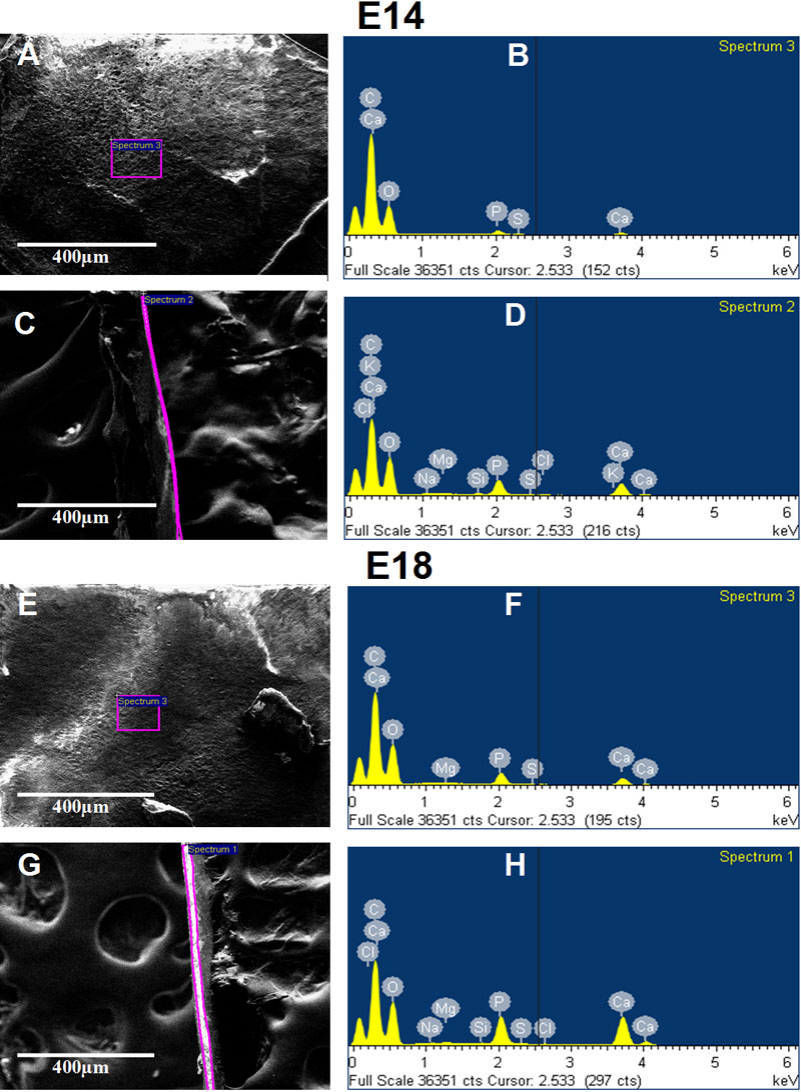
Representative FESEM select area EDS analysis of chicken ossicles. Surface (**A**, **E**) and interior (**C**, **G**) secondary electron images and corresponding spectrum (**B**, **D**, **F**, and **H**) are presented from two different developmental time periods E14 (**A**, **B**, **C**, and **D**) and E18 (**E**, **F**, **G**, and **H**). The regions outlined in magenta in the images indicate selected areas that were analyzed by EDS; corresponding spectra are presented to the right. Scale bar, 400 µm.

**Figure 9:**
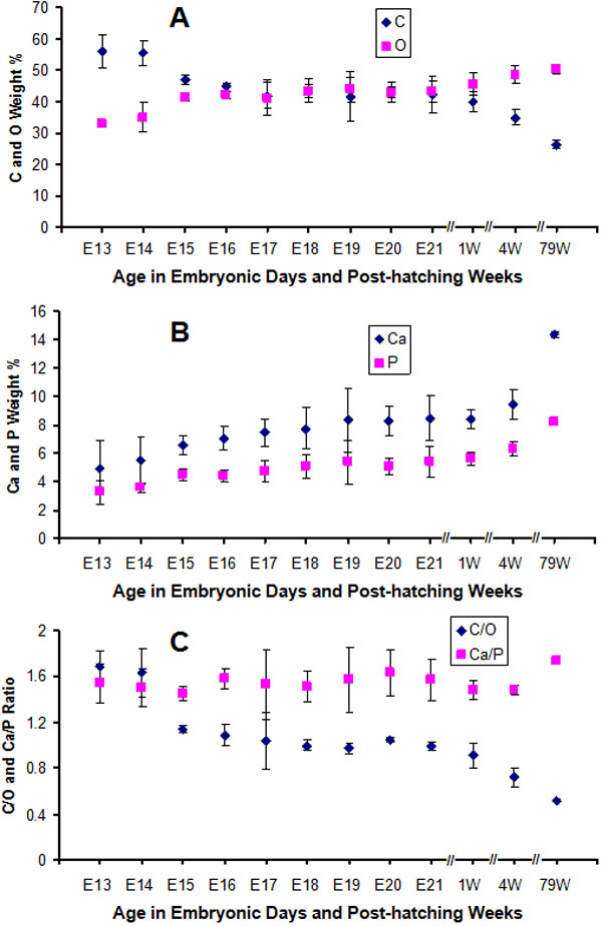
Quantification of the mineralization of chicken ossicles. Data were derived from the interior region of each ossicle, as viewed in cross-section (Figure 7) **A**: Patterns of C and O in chicken ossicles during development; **B**: Patterns of Ca and P in chicken ossicles during development; **C**: C/O and Ca/P ratios in chicken ossicles during development. Crosshatch gaps (//) are shown between the E21 and 1 week ages, and between the older ages to emphasize that the time scales in these 3 regions are not a linear continuous line.

Data in Figure 8 represent FESEM select area EDS analysis of chicken ossicles. The red-purple-outlined regions in the images indicate the selected areas that were analyzed by EDS and presented in corresponding spectra to the right. Figure 9 shows the concentrations of the main elemental components (C, O, Ca, and P) in chicken scleral ossicles during embryonic stages E13 to E21 embryos, and in post-hatching 1-week-old chickens, 4-week-old chickens, and 79-week-old adult chickens. These data show that carbon (C) levels decrease from E14 to E18, and continue decreasing even until 79 weeks post-hatching (Figure 9A), while oxygen (O) levels increased during those same periods (Table 1, Figure 9A). Calcium levels in ossicle interior regions were always higher than levels on the ossicle surface. The concentrations of both Ca and P (Figure 9B) steadily increased during embryogenesis and post-hatching, the period of overt osteogenesis in the scleral ossicles. Therefore, in contrast to the C/O ratio (Figure 9C) which declined during this period, the Ca/P ratio remained relatively constant, suggesting the steady accumulation of bone of constant composition, even as the total mass of that bone increased during embryonic development and post-hatching.

## Discussion

In this study, we developed a simple, rapid, sensitive, consistent technique to visualize developmental morphology and monitor the mineralization of avian scleral ossicles using Giemsa staining and FESEM/EDS. Giemsa bone staining is more sensitive than Alizarin Red S, allowing earlier detection of osteogenesis during development (compare Giemsa staining in Figure 1 at E12 to Alizarin Red S staining in Figure 2 at E14). Moreover Giemsa-stained tissue provides more details of bone substructure (lacunae, Figure 4) and is compatible with fluorescent nuclear stains (Figure 6). In addition, FESEM/EDS analysis allows rapid quantification and localization of ossicle mineralization, providing detailed characterization of membrane bone differentiation and development. FESEM/EDS analysis is a powerful technique that could be used to detect and quantify the presence of C, O, Ca, and P (as well as trace elements, data were not shown here) in two spatially distinct domains (the surface and interior) of individual chicken ossicles.

To our knowledge, the embryonic morphology and development of scleral ossicles in Japanese quail had not been described previously. One earlier publication described a morphological analysis of the scleral ossicles and their modal number, but only in ages of adult quail [10]. Using Giemsa fluorescence staining we found that the detailed structure and developmental morphology of quail ossicles (Figure 3) were remarkably similar to those of chickens (Figure 1). Prominent preossicular condensations were detected as early as E10 in chickens and E8 in quail, while the first sign of overt ossification appeared by E11 in chickens. The results demonstrate that quail scleral ossicle development closely resembles that of chickens. These data support previous reports that ossification first occurs in chickens by E11 and that thin ossicle plates are present at E12 [3,4,22]. In comparison, the earliest ossification detected in scleral ossicles of Japanese quail embryos was seen by E9, and a ring of ossicle plates was visible by E12 (Figure 3).

In birds, scleral ossicles are neural crest-derived membrane bones [23,24]. Neural crest cells migrate into the head region, proliferate, and give rise to the mandibular and maxillary processes and to the mesenchyme surrounding the eye. After migration into the region surrounding the cornea, these cranial neural crest cells encounter diverse extracellular environments, including those beneath specialized papillae that form in a ring in the conjunctival epithelium (conjunctival papillae). Each conjunctival papilla induces the formation of a single scleral ossicle in the underlying mesenchyme [2,22,23,25-27]. In chicken embryos, this scleral skeleton normally comprises 14 ossicles and develops during the second week of incubation following a prolonged and intimate association with the transitory conjunctival papillae. The conjunctival papillae arise during E6-E8 of development, and by E8 the maximum number of 13–14 papillae has been formed. This corresponds precisely to the number of areas of underlying scleral mesenchyme in which ossicles subsequently form. In chickens, the papillae undergo a complex pattern of morphogenesis in intimate association with underlying subjacent pre-scleral mesenchyme tissue. By E11 of development the papillae have degenerated, just as overt bones begin to form [26,27].

Osteocytes are the most abundant cells in bone and the only cells embedded in the calcified bone mineral matrix [28], occupying small cavities called osteocyte lacunae. Osteocytes are connected with neighboring cells by means of dendritic cell processes [29]. Evidence presented in Figure 4 indicates that the microporosities documented in chicken scleral ossicles appear by E15 and their density increases until E21, during the development of the ossicle plate, suggesting that faster growth of ossicle tissue begins around E15 and continues until hatching. After hatching, microporosity density does not appear to increase further between E21 and 4 weeks post-hatching (Figure 4). As shown in Figure 6, each individual bone microporosity contains a nucleus, presumably that of an individual osteocyte, revealing that the osteocyte density increases that are seen in Figure 4 are consistent with the steady ossicle mineralization documented in Figure 7, Figure 8, and Figure 9. To our knowledge, this progressive increase in microporosity (i.e., in the density of the lacunae housing individual osteocytes in scleral ossicles) during embryogenesis has not been demonstrated previously. That increase may simply reflect the progressive expression of osteocyte differentiation by proliferating, but initially non-differentiated, neural crest cells present in the preossicular condensations.

The increased staining intensity using Giemsa fluorescence with increasing developmental age is consistent with data presented in Figure 5, Figure 6, Figure 7, and Table 1, providing supportive evidence and new insights into the changes in development and mineralization of chicken scleral ossicles. Our data demonstrate that the degree of mineralization in the bone matrix in the internal region of individual scleral ossicles is significantly and consistently higher than that on the surface throughout development, suggesting that the deposition of Ca and P in hydroxyapatite mainly occurs deep within each scleral ossicle.

Crystalline carbonated hydroxyapatite, an inorganic calcium phosphate mineral, constitutes a substantial component of mature bone tissue synthesized by osteocytes [30,31]. The mineralization and mechanical properties of bone largely depend upon the deposition levels of Ca and P [20,32]. The data presented in Figure 9 demonstrate that the concentrations of Ca and P increase steadily from E13 in embryogenesis, past hatching and into adulthood. In contrast, during this same period, the concentrations of C decline steadily and the levels of O increase. These changes in composition reflect the increasing mineralization of scleral ossicles. Furthermore, the relatively constant (1.5) Ca/P ratios (Figure 9C) suggest that the composition of the scleral ossicle interior region remains approximately constant from embryogenesis to adulthood.

In conclusion, the work presented here represents a novel approach for studying the development and mineralization of avian scleral ossicles. Details and entire features of the developmental morphologies of both chicken and quail ossicles were documented using Giemsa as a very rapid fluorescent stain for ossicle development and mineralization. Moreover, FESEM and EDS supported Giemsa staining findings concerning the presence of a calcified matrix and expanded our quantitative and qualitative knowledge of mineralization within discrete regions of avian scleral ossicles related to developmental stages.

## Appendix 1. The patterns of chicken ossicle development according to their positions in the ossicle ring.

**A**: The temporal ossicles at ages E10 to E21. **B**: The nasal ossicles at ages E10 to E21. **C**: The ventral ossicles at ages E10 to E21. The details of dorsal ossicles were shown in Figure 1B. Scale bar: 2 mm. To access the data, click or select the words “Appendix 1.” This will initiate the download of a compressed (pdf) archive that contains the file.
